# Identifying surge of exclusionary nationalism: A case study of prewar Japan

**DOI:** 10.1371/journal.pone.0349895

**Published:** 2026-07-08

**Authors:** Tomoko Matsumoto, Yutaka Shimada, Hiroyuki Hirate, Tohru Ikeguchi

**Affiliations:** 1 Institute of Arts and Sciences, Tokyo University of Science, Shinjuku-ku, Tokyo, Japan; 2 Graduate School of Science and Engineering, Saitama University, Saitama, Japan; 3 Department of Information and Computer Technology, Tokyo University of Science, Katsushika-ku, Tokyo, Japan; Universidade Federal do Tocantins, BRAZIL

## Abstract

A rise in exclusionary nationalism can escalate conflicts; however, there are limited quantitative analyses of the timing and magnitudes of such shifts. This study proposes a quantitative approach for tracking changes in exclusionary nationalism by focusing on language, specifically the acceptance/rejection of foreign loanwords. Using 300,110 Japanese newspaper articles spanning 1912–1943—the period surrounding the outbreak of the 1941 conflict with the United States and the United Kingdom—we employed the singular spectrum transformation method to detect change points in exclusionary nationalism. We found that exclusionary nationalism emerged in Japan in 1936 and subsequently exhibited substantial oscillations rather than continuous intensification in the years leading up to the outbreak of the Pacific War. Moreover, attitudes toward potential adversaries and allies were evident from the 1920s. This study deepens our understanding of the role of exclusionary nationalism in conflicts, highlighting its nuanced nature in shaping friend–enemy distinctions during wartime.

## Introduction

Nationalism has re-emerged as a powerful force shaping contemporary politics and international relations. While the end of the Cold War was widely viewed as the triumph of globalization and the decline of nationalist ideologies [1], the persistence—and resurgence—of nationalism in recent decades challenges this expectation. From the ethnic conflicts in the Balkans and the former Soviet Union during the 1990s to the rise of exclusionary politics, including far-right movements in developed nations, nationalism continues to reshape global and domestic power dynamics. The COVID-19 pandemic further intensified these trends [2,3], while the ongoing Ukrainian conflict underscores the relationship between nationalism and international tensions.

The association between national attachment and international cooperation has been a long-debated topic in political science [4]. Liberal nationalism advocates argue that patriotism encourages support for international collaboration [5–9]. However, due to the need to define the group boundaries inherent in group affiliation, many other scholars remain skeptical of this positive relationship [10–13]. In this regard, psychologists distinguish nationalism from patriotism. Nationalism is the ideological belief that one’s own nation should be superior to others [14]. This study follows this definition and uses the term *exclusionary nationalism* to emphasize that our focus is on nationalism with an exclusionary nuance.

Despite this ongoing debate, scholars, including those aligned with liberal nationalism, have largely agreed that, if patriotism evolves into exclusionary nationalism, its impact on international cooperation becomes detrimental [15], which increases the risk of conflict escalation toward war. However, it remains unclear whether exclusionary nationalism in a country on the verge of war is directed at all countries indiscriminately or targets certain nations as potential adversaries [16]. Cottam [17] and Herrmann [18] suggest that attachment to a nation leads to positive images of functional allies and negative images of functional foes. Hence, the surge in exclusionary nationalism may foster exclusionary sentiments toward potential enemy countries while not necessarily impacting attitudes toward potential allies.

Furthermore, as Tilly posited that “war made the state and the state made war,” warfare often fuels exclusionary nationalism [19]. A comparison of the magnitude of the change on the eve of a war with that at the onset would show whether the rise in exclusionary nationalism is the cause or the influence of war. If a rise in exclusionary nationalism causes war [4], this rise starts on the eve of war and its magnitude is greater than that at the onset of the war; conversely, if a war increases exclusionary nationalism, the rise in exclusionary nationalism is observed following war onset. Therefore, assessing the onset and extent of exclusionary nationalism is crucial for understanding the causal link between warfare and exclusionary nationalism. However, apart from using public opinion polls, quantitative analyses that track the timing and magnitudes of these shifts are limited.

In light of these considerations, this study develops and applies a statistical approach to detecting salient shifts in exclusionary nationalism in newspaper discourse before and after the outbreak of the war. Rather than adjudicating causal primacy between war and exclusionary nationalism, our aim is to identify when and how exclusionary nationalist language becomes discursively salient in the period surrounding major military conflict. Therefore, we focus on language use in newspapers as an observable trace of exclusionary nationalist dynamics. To capture exclusionary nationalism from observational data, this study focuses on language as the fundamental communication tool for humans to exchange information and thoughts [20]. Scholarly discourse has extensively explored the relationship between language and nationality [21], and a few recent research endeavors have sought to quantify nationalism using text data and the affordances of neural language models [22]. However, quantifying exclusionary nationalism has been deemed intricate, given that the terminology used to articulate exclusionary nationalism tends to evolve over time, possesses semantic polysemy, and exhibits diversity. Moreover, assessing the extent to which exclusionary nationalism aligns with hostility toward a specific country has been demanding.

To address these challenges, we propose a new approach that considers the utilization of foreign loanwords. The historical nexus between nationalism and foreign loanwords provides a clear lens through which to examine this relationship. One of the most historically well-known examples showing this connection is British anti-Germanism during the First World War [23–25]. Soon after the onset of the war, the British government and people removed the use of German-related words from the country. For instance, Berlin Road in Catford, London, was renamed Canadian Avenue, and the German biscuit was renamed the Empire biscuit. Under increasing domestic pressure, King George V even changed his German name of Saxe-Coburg and Gotha to Windsor in 1917. This exclusion of “enemy language” is not unique to the United Kingdom. In January 2022, shortly before Russia’s February 24 invasion, Ukraine introduced a new restriction law under which print media outlets registered in Ukraine were required to publish in Ukrainian, with several exceptions, such as English and other official European Union languages. This law intended to prohibit print media outlets from publishing in Russian. Other countries and media companies that had criticized the Russian invasion also followed this trend, although to a lesser extent, for example, by eliminating the Russian spellings of Ukrainian cities. The spelling of the capital city of Ukraine changed from the Russian-derived “Kiev” to “Kyiv,” a transliteration of the Ukrainian spelling. These two cases suggest that, when we use language, we are consciously or unconsciously aware of the country from which a word originates. Therefore, a rise in exclusionary nationalism is expected to influence people’s word choices. Specifically, as exclusionary nationalism increases, people become more likely to use words originating from their own language over foreign words.

**Fig 1:**
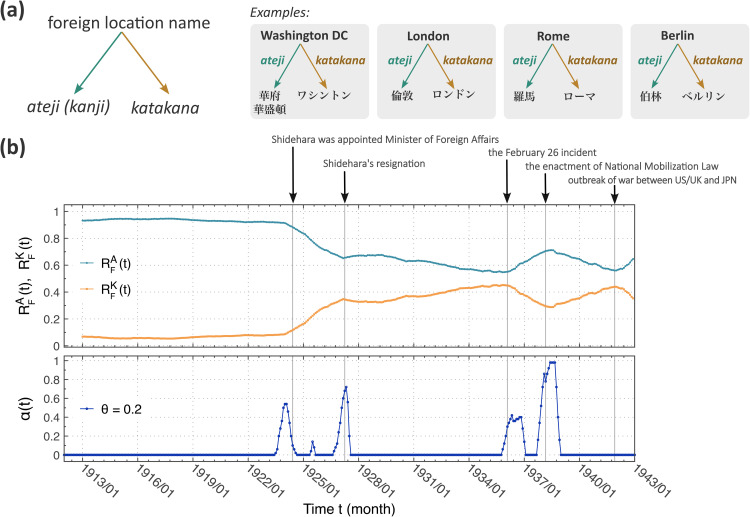
Examples of *ateji* using *kanji* and *katakana* for foreign location names and the ratio of the number of *ateji* words referring to location names located in all foreign countries to the number of both *ateji* and *katakana* words referring to them at time *t* ($R_{𝐅}^{𝐀}(t)$). **(a)** Examples of *ateji* and *katakana* representations of several capital cities. **(b)** The upper part of the figure shows the *ateji* ratio at time *t* ($R_{F}^{A}(t)$). $R_{F}^{K}(t)\;(=1-R_{F}^{A}(t))$ is also plotted to compare i*t* with the *ateji* ratio. The bottom part of the figure shows the variation score α(t) obtained by an SST-based method (θ=0.2).

To demonstrate the effectiveness of our methodology, this study explores the frequency of using foreign loanwords in a large dataset of 300,110 articles published by 54 Japanese newspapers before and after the onset of the Japanese war against the United States and the United Kingdom in 1941. The case is advantageous because Japanese facilitates the easy distinction between foreign loanwords and native words through the distinct usage of characters. In the Japanese language, there are three different types of characters: *hiragana*, *katakana*, and *kanji*. *Katakana* is a Japanese syllabary mainly used to transcribe foreign-language words into Japanese, while *hiragana* and *kanji* are used for traditional Japanese words. During the early westernization phase of the late 19th century, the Japanese chose to create a new Japanese word called an *ateji* using *kanji* for foreign location names. Afterward, people gradually preferred foreign loanwords using *katakana*. Consequently, they could choose either a foreign-language *katakana* loanword, its translated Japanese *kanji* word, or *ateji* when mentioning a location name in a foreign country (Fig 1(a)). This unique characteristic allows us to observe changes in exclusionary nationalism and differences in exclusionary attitudes toward ally and enemy countries before and after the onset of the war in December 1941. We employ the singular spectrum transformation (SST) method to detect change points in the time series of the frequency of using foreign loanwords. By doing that, we identify which countries’ loanwords became more or less prevalent in newspaper discourse, as well as the timing of these shifts, thereby capturing key temporal patterns in the emergence of exclusionary nationalism in public media.

## Materials and methods

### Data

This study explores the Japanese war against the United States and the United Kingdom in 1941. This case is advantageous for testing the detection method of exclusionary nationalism for the following reasons. First, the Japanese government’s decision to declare war against the two countries cannot be explained by rational calculations of costs and benefits. Many reports of those days predicted that Japan would have great difficulty in fighting the United States and the United Kingdom, a view widely shared by politicians, the military, and intellectuals; the press even highlighted the huge power gap between these two countries and Japan [26]. Therefore, exclusionary nationalism was expected to play a significant role in this war. Second, as opposed to other non-Western countries, Japan achieved westernization and even joined the League of Nations in 1920 as one of four permanent League Council members. Before the war, Japan saw itself as a world leader and maintained an open attitude toward other countries. Furthermore, since the regime change in 1868, it was not involved in any war against the United States or the United Kingdom prior to 1941. Hence, the rise of exclusionary nationalism was required to change people’s international mindset toward prosecuting the war. Third, there were several potential turning points leading to the escalation of exclusionary nationalism during the period [27,28]. These include the 1932 coup attempt by a faction of the Japanese military, known as the May 15 incident; Japan’s withdrawal from the League of Nations in 1933; another coup attempt in 1936, referred to as the February 26 incident; the enactment of the National Mobilization Law in 1938; the signing of the Tripartite Pact between Japan, Germany, and Italy in 1940; and the attack on Pearl Harbor in 1941.

To analyze the change in exclusionary nationalism in Japan before and after the onset of the war in December 1941, we utilized newspaper articles published in Japan during that time (see also Table S2 in S1 File for examples of newspaper articles). The most desirable way to scrutinize shifts in nationalism within public opinion would be to use public opinion polls [29], but this method encounters limitations due to the specific timeframes and countries in which the polls were conducted. Consequently, there is a dearth of quantitative studies on exclusionary nationalism beyond Western countries in recent periods, with few exceptions, such as Karasawa [30]. The utilization of media coverage becomes crucial in this context, as it allows consideration of broader timeframes and geographical regions.

It is important to acknowledge that media perspectives may not invariably align with public opinion. In fact, the media and public opinion are inherently symbiotic, as the media exerts its influence over agenda setting and framing while also responding to both formal and informal pressures from the public. Furthermore, the media may also be susceptible to governmental influence [31], especially on the eve of and during wars. In Japan, under the 1909 Newspaper Law and the 1925 Peace Preservation Law, newspapers were subject to censorship and could, in some cases, be suspended. In addition, following the enactment of the National Mobilization Law in 1938, newspaper publishers faced increasingly stringent state control. These controls ensured that their publications reflected not only society’s but also the government’s intentions during our observed period, especially after the mid-1930s [32]. According to Oishi [33], the movement of rejecting English words gradually started four years before the outbreak of the war in 1941 because of growing frustration with the United States and, to a lesser extent, the United Kingdom, which had criticized Japanese aggression toward China in 1937. Nevertheless, no formal law prohibited English words, although there were sporadic measures in 1940, such as the Ministry of Education’s prohibition of Anglo-American-style school names and the Ministry of Railways’ ban on English signage within railway stations. Although opportunities for newspapers to mention overseas place names may have declined as censorship intensified, this reduction is analytically distinct from—and does not bear on—the question of how such place names were rendered when they did appear. That is, variation in the frequency of overseas references affects the volume of observations, whereas our analysis focuses on orthographic choice (*katakana* versus *ateji*) conditional on mention, which remained formally unconstrained by explicit government directives.

Beyond editorial and ideological considerations, it is also necessary to examine whether technical and typographical constraints may have influenced orthographic choices in newspaper representations of foreign place names. When considering the choice between *katakana* transliterations and *ateji* in the representation of foreign place names, one technical consideration is that *katakana* forms tend to require a greater number of characters. However, there is no evidence that Japanese newspapers altered basic layout conventions—such as reducing the number of characters per line—during the period under observation (1912–1943), nor did they depart from the standard practice of vertical typesetting. This consistency suggests that typographical constraints are unlikely to account for systematic variation in orthographic choice.

To collect newspaper articles from prewar Japan, we contacted Kobe University and utilized the Newspaper Clipping Collections created by their Research Institute for Economics and Business Administration (http://www.lib.kobe-u.ac.jp/sinbun/e-index.html). The dataset was extracted in November 2020. This collection covers mainly the commercial economy but also various other topics, such as politics, culture, society, and law. Moreover, they clipped more than 50 newspapers, which helped mitigate any potential bias. The number of target articles was small in 1911 and dropped significantly after 1943. Therefore, we focused on data from 1912 to 1943. The total number of newspaper articles in our dataset was 300,110 from 54 newspapers. The procedure for making a list of *katakana* and *ateji* foreign location names is reported in Sec. S1 in S1 File (see also Sec. S2 in S1 File for details of the number of newspaper articles in each year and month).

### *Ateji* and *katakana* ratios

Our focus is on the frequency of using *kanji* (i.e., *ateji*), rather than *katakana*, for foreign location names. In addition to all foreign locations except Japan, we targeted four countries: the United States and the United Kingdom, as the major potential Japanese enemy countries at that time, and Germany and Italy, as the major potential allies.

We mainly investigated month-by-month changes in the following ratio: the ratio of the number of *ateji* words referring to location names located in country “X” to the number of both *ateji* and *katakana* words referring to them at time *t*, defined by:


$$\begin{array}{c} R_{X}^{A}(t)=\frac{P_{X}^{A}(t)}{P_{X}(t)}, \end{array}$$ (1)

where $P_{X}^{A}(t)$ is the number of *ateji* words referring to location names located in country X at time *t* and $P_{X}(t)$ is the number of both *ateji* and *katakana* words referring to them at time *t*. In Eq. (1), the unit of time is one month. This study focuses on four countries: Germany, the Uni*t*ed Kingdom, Italy, and the United States. Thus, X ∈ {F,DEU,GBR,ITA,USA}, where “F” denotes all foreign countries and “DEU,” “GBR,” “ITA,” and “USA” denote Germany, the United Kingdom, Italy, and the United States, respectively. We call the ratio defined by Eq. (1) the *ateji ratio* throughout the paper. In addition to the *ateji* ratio, we also define the following ratio with respect to *katakana* words:


$$\begin{array}{c} R_{X}^{K}(t)=\frac{P_{X}^{K}(t)}{P_{X}(t)}, \end{array}$$ (2)

where $P_{X}^{K}(t)$ is the number of *katakana* words referring to location names located in country X ∈ {F,DEU,GBR,ITA,USA} at time *t*. To smooth the time series of these ratios, we calculated the moving average over 13 mon*t*hs from January 1, 1912, to December 31, 1943. The moving average of *r*(*t*) is calculated as $\bar{r}(t)=\frac{1}{13}\left\{r(t)+\sum_{\tau =1}^{6}\left[r(t+\tau )+r(t-\tau )\right]\right\}\;(6<t<T-6)$, where r(t) (t=1,2,…,T) is one of the ratio time series.

### How to detect pivotal turning points

The *ateji* ratio allows us to observe changes in the collective social behavior of Japanese people because articles in major newspapers can reflect or influence public opinion. Therefore, the *ateji* ratio and the *katakana* ratio can be good indicators of temporal changes in Japanese public opinion, as significant changes in these ratios might reflect changes in public opinion in Japan. To detect such changes, we used a typical change-point detection method, the SST [34], which is based on singular spectrum analysis [35]. The SST does not require a generative model and can detect change points in a time series almost without prior information about the time series. Using the SST is thus one of the promising approaches to change-point analysis of real time series. Utilizing the SST, we estimated the variation score α(t) of the time series of the ratios for each temporal point as follows.

Given a time series of ratio r(t) (t=1,2,…,T), we used the following algorithm to examine whether each temporal point is a change point of the time series *r*(*t*) by the SST:

1. Define vector $x(t)≔(r(t),r(t+1),\ldots ,r(t+M-1){)}^{⊺}\;(t=1,2,\ldots ,T-M+1)$ and construct an M×n matrix *X*(*t*) called a *trajectory matrix* by X(t)=[x(t−n−M+1),x(t−n−M+2),…,x(t−M)], where ^⊺^ denotes the transpose of a vector or a ma*t*rix, *n* is a parameter for determining the number of columns of *X*(*t*), and *M* is a parameter for determining the number of rows of *X*(*t*).
2. Construct an M×k matrix *Z*(*t*) called a *test matrix* by Z(t)=[x(t−k+ℓ−M+1),x(t−k+ℓ−M+2),…,x(t−M+ℓ)], where *k* is a parame*t*er for determining the number of columns of *Z*(*t*) and ℓ is a parameter for controlling the time gap between the trajec*t*ory matrix and the test matrix. We set *n* = *k* and ℓ=M+k (=M+n).
3. Apply the singular value decomposition (SVD) to *X*(*t*) and generate a matrix $U_{m}^{\left(t\right)}$ by $U_{m}^{\left(t\right)}≔(u_{1}^{\left(t\right)}\;u_{2}^{\left(t\right)}\;\ldots \;u_{m}^{\left(t\right)})$, where $u_{i}^{\left(t\right)}\;(i=1,\ldots ,m)$ is the left singular vector corresponding to the *i*th largest singular value, and *m* is the number of nonzero singular values of *X*(*t*).
4. Apply the SVD to *Z*(*t*) and generate a matrix $Q_{m^{\prime }}^{\left(t\right)}$ by $Q_{m^{\prime }}^{\left(t\right)}≔(q_{1}^{\left(t\right)}\;q_{2}^{\left(t\right)}\;\ldots \;q_{m^{\prime }}^{\left(t\right)})$, where $q_{i}^{\left(t\right)}\;(i=1,\ldots ,m^{\prime })$ is the left singular vector corresponding to the *i*th largest singular value, and $m^{\prime }$ is the number of nonzero singular values of *Z*(*t*).
5. Calculate the change-point (CP) score at temporal point *t* [36], denoted by *c*(*t*), as:

$$\begin{array}{c} c(t)=1-\|{U_{m}^{\left(t\right)}}^{⊺}Q_{m^{\prime }}^{\left(t\right)}{\|}_{2}, \end{array}$$ (3)

where $\|{U_{m}^{\left(t\right)}}^{⊺}Q_{m^{\prime }}^{\left(t\right)}{\|}_{2}$ is the *L*_2_ norm of the matrix ${U_{m}^{\left(t\right)}}^{⊺}Q_{m^{\prime }}^{\left(t\right)}$. If the CP score is higher than a predetermined threshold, the candidate change point *t* is considered to be a change point.

In the SST, when examining whether temporal point *t* is a change point of the time series *r*(*t*), we compare two subsequences of *r*(*t*), namely {r(t−n−M+1), r(t−n−M+2), …, r(t−1)} and {r(t+1), r(t+2), …, r(t+M+n−1)}, and thus *M* and *n* are the control parameters for determining the size of the time windows for these subsequences. Selecting the parameter values of *M* and *n* is important because the values of *M* and *n*, namely the size of the time windows, significantly affect change-point detection results. To detect reliable change points, we repeatedly performed the SST with multiple pairs of parameter values of *M* and *n*. We then consistently extracted the detected change points even when the parameter values of *M* and *n* changed. The following procedure shows how to extract these change points.

1. Let η be a set of possible pairs of parameter values of *M* and *n* such that *M* and *n* satisfy 6≤M≤12 and 6≤n≤12.
2. Apply the SST to the time series $\bar{r}(t)$ for all pairs of parameter values (M,n)∈η and t∈(M+n−1,T−M−n). Then, compute the CP score, $c_{M,n}(t)$.
3. Normalize the CP scores as:
  $$\begin{array}{c} c_{M,n}^{\prime }(t)≔\frac{c_{M,n}(t)}{\max_{t}c_{M,n}(t)}, \end{array}$$ (4)
  where $c_{M,n}^{\prime }(t)$ denotes the normalized CP score.
4. Let κ(t) be the number of times $c_{M,n}^{\prime }(t)$ exceeds a threshold θ for all pairs of (*M*,*n*) in η. Then, the variation score is calculated for temporal point *t* by α(t)≔κ(t)/|η|, where |η| is the number of elements in set η.

The variation score is the rate at which temporal point *t* is detected as a change point; thus, temporal point *t* is considered to be a plausible change point when the value of α(t) is high.

The SST can detect small change points in the time series when the value of *M* and/or *n* is small, which implies that the SST with a small time window tends to be affected by noise. In step 1, to avoid the influence of noise, we set the minimum values of the ranges of *M* and *n* to six. Moreover, to detect changes in the ratio time series over several years, we set the maximum values of the ranges of *M* and *n* to 12, and the time window size (*M* + *n*) is constrained to be larger than or equal to 12. In step 4, we set the threshold θ to 0.2. Note that even if the threshold slightly changes, the obtained results do not change substantially (see also Sec. S3 in S1 File for details).

## Results

Using the dataset and the SST, we quantitatively substantiate the changes in exclusionary nationalism and their magnitudes. Specifically, this study examined changes in the transcription of foreign geographical designations in newspaper articles. As Fig 1(a) illustrates, there are two notations, *ateji* and *katakana*, for every foreign location name. We calculated the frequency of using *ateji* when referring to geographical locations in each foreign country, interpreting this as an indicator of exclusionary attitudes toward that country. We first investigated the month-by-month changes in the *ateji* ratio, $R_{X}^{A}(t)$, and the *katakana* ratio, $R_{X}^{K}(t)$, defined in Eqs. (1) and (2), where X ∈ {F,USA,GBR,DEU,ITA} and “F” denotes all foreign countries as introduced in the Materials and methods section.

Fig 1(b) illustrates both the *ateji* ratio and the *katakana* ratio in the upper panel, along with the identified change points estimated by the SST-based method in the bottom panel. The variation score α(t) of the ratios for each temporal point in the bottom panel of Fig 1(b) shows that a temporal point *t* can be a plausible change point if the variation score α(t) a*t* that temporal point is high (as detailed in the Materials and methods section). Therefore, in the bottom panel of Fig 1(b), the points where the variation score α(t) exhibits significant peaks can be interpreted as pivotal turning points. When analyzed chronologically, notable peaks are observed around 1924, 1927, 1936, and 1938, where these years are closely related to the following historical events (see [37–39] for the detailed historical narratives).

The increase in the *katakana* ratio reflects the rise of internationalism since the 1920s. The first significant turning point occurred in 1924, when the *ateji* ratio began to decrease, subsequently leading to an increase in the *katakana* ratio. In 1924, Kijūrō Shidehara was appointed Minister of Foreign Affairs, marking a significant moment during the interwar period characterized by international cooperation in Japan’s diplomatic efforts. His appointment came amid the Second Constitutional Protection Movement, which arose in early 1924 to criticize aristocratic-dominated cabinets and demand universal suffrage, and followed the May general election that brought the party-based cabinet to power. In his inaugural speech, Shidehara declared his commitment to upholding the Versailles-Washington system, which served as a catalyst for growing international cooperation within Japanese society. The fact that the period during which the *katakana* ratio increases coincides with the timing that Shidehara was appointed Minister of Foreign Affairs leads us to expect that the increase in the *katakana* ratio can be interpreted as the strengthening influence of internationalism, although we must also consider other factors. Furthermore, the deceleration in the increase rate of the *katakana* ratio in 1927 aligns with Shidehara’s resignation.

To identify pivotal junctures signifying the rise of exclusionary nationalism, we initially focused on key inflection points marked by a decline in the use of *katakana* and a concurrent increase in the utilization of *ateji* when mentioning foreign places. Our analysis from 1912 to 1943 revealed a surge in the *ateji* ratio in 1936, suggesting a rise in exclusionary nationalism.

The year 1936 is renowned for the February 26 incident—an attempted coup d’état led by young military officers with the aim of overthrowing the government by citing various grievances, including their perception of governmental leniency, particularly toward liberal principles. In pursuit of their goals, they assassinated prominent figures, although the intervention of Emperor Hirohito ultimately quelled the coup. Consequently, this event left an enduring mark on Japanese politics, resulting in heightened militarization and the ascendancy of radical elements within the military.

This highlights two intriguing aspects. First, there was a gap between the rise of the *ateji* ratio and Japan’s withdrawal from the League of Nations in 1933. Second, this critical juncture occurred significantly earlier than the enactment of the National Mobilization Law in 1938 and the outbreak of the war between the United States / the United Kingdom and Japan in December 1941. In fact, it was the second, not the first, increase in the *ateji* ratio that aligned with the outbreak of the war between the United States / the United Kingdom and Japan in December 1941.

It is also noteworthy that the *ateji* ratio did not consistently increase from 1936 to 1941. Rather, it decreased soon after the enactment of the National Mobilization Law in 1938, which may suggest that, even after the law’s implementation, there were efforts to avoid a conflict with Western countries.

**Fig 2:**
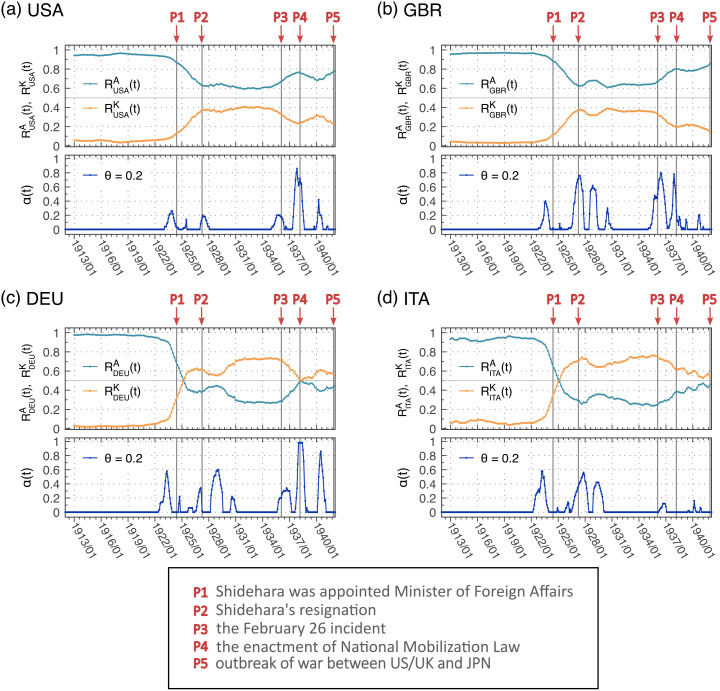
The *ateji* ratios for Japanese ally and enemy countries during the war. **(a)**–**(d)** show the *ateji* ratio ($R_{X}^{A}(t)\;(X\in \{DEU,GBR,ITA,USA\}$). $R_{X}^{K}(t)\;(=1-R_{X}^{A}(t))$ is also plotted to compare it with the *katakana* ratio (see also Sec. S4 of S1 File for examples of *ateji* and *katakana* ratios of country names corresponding to DEU, GBR, ITA, and USA for reference). The bottom figures show variation scores α(t) obtained by applying the SST to the time series of the *ateji* ratio for each country.

The increase in the *ateji* ratio and the decrease in the *katakana* ratio suggest the rise of exclusionary nationalism and the decline of internationalism. However, it is unclear from Fig 1(b) whether the heightened attitude was directed toward specific countries or all other nations. To address this issue, Fig 2 shows the fluctuation in the *ateji* ratio in the location names of Japan’s major adversary nations, (a) the United States and (b) the United Kingdom, as well as the major ally nations, (c) Germany and (d) Italy, during the Pacific War.

The plots in Fig 2 demonstrate a trend similar to that in Fig 1(b). However, upon closer examination, when comparing attitudes toward future enemy countries, the United States and the United Kingdom, with those toward future ally countries, Germany and Italy, a significant distinction emerges as early as the 1920s, well before the onset of the Pacific War. While there was no notable discrepancy in the *ateji* ratio among these countries until the 1910s, the decline of the *ateji* ratio became apparent in the 1920s. During this decade, although the *ateji* ratio decreased for American and British location names, it remained higher in frequency than *katakana*. Conversely, for German and Italian location names, the use of *katakana* began to surpass that of *ateji*. This disparity persisted even when exclusionary nationalism surged in 1936. While the use of *ateji* increased uniformly when referencing any country, the *katakana* ratio never dropped below the *ateji* ratio for Germany and Italy.

Moreover, we observed differences even among enemy and ally countries. Considering Germany as a major potential ally and the United States as a major potential enemy, after an increase in the *ateji* ratio in 1936, the *katakana* ratio surged notably again in 1938. This can be attributed to simultaneous efforts to avoid a conflict with the United States and strengthen the cooperative relationship with Germany on the eve of the war. By contrast, such a backlash was scarcely observed for the United Kingdom and Italy, as both experienced a gradual expansion of the *ateji* ratio after 1936.

**Fig 3:**
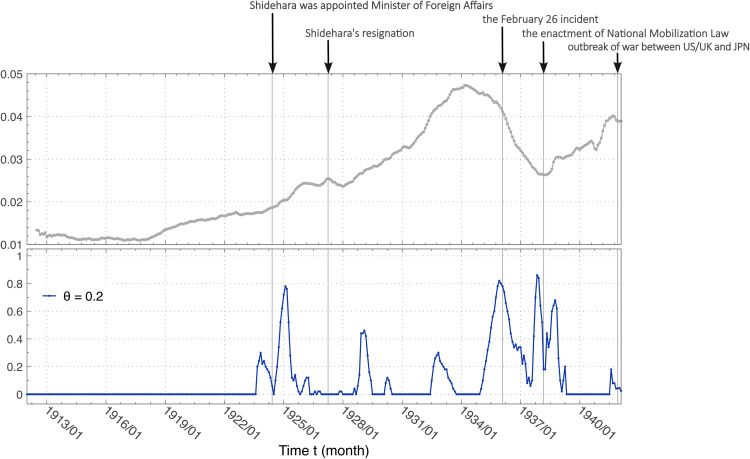
The upper figure shows the ratio of the number of all *katakana* words to the number of all words in articles ($\bar{K}(t)$), and the bottom one shows the variation score α(t) obtained by applying the SST to the time series of $\bar{K}(t)$ (θ=0.2).

To ensure the robustness of the findings in Fig 1(b), we conducted an additional analysis of the shifts in the proportion of *katakana* words within our dataset, given that *katakana* words predominantly represent foreign terminology. We calculated the ratio of the number of all *katakana* words to the number of all words in articles, which is denoted by *K*(*t*), and report the results in Fig 3, which shows a similar pattern to Fig 1(b).

## Discussion

One of the central findings of this study is that exclusionary nationalism in prewar Japan did not increase monotonically as the country approached the outbreak of the Pacific War in 1941. Instead, the time series exhibits pronounced oscillations, including periods of both intensification and retreat, indicating that the discursive trajectory of exclusionary nationalism was neither linear nor inevitable. Importantly, these shifts unfolded during a period already marked by ongoing military conflicts and international tensions, rather than emerging in the absence of conflict-oriented events.

Using SST on Japanese newspaper data from 1912 to 1943, we show that Japan had already experienced a surge in exclusionary nationalism in 1936, marked by the February 26 Incident, several years before the outbreak of the war in 1941. Importantly, rather than continuing to intensify thereafter, exclusionary nationalism fluctuated substantially between 1936 and 1941.

Moreover, attitudes toward foreign countries did not simply crystallize at the onset of war or as a direct consequence of heightened exclusionary nationalism. Distinct patterns were already evident in the 1920s: attitudes toward the United States and the United Kingdom—later enemy nations—differed systematically from those toward Germany and Italy, which later became allies. These differences persisted even during periods of elevated exclusionary nationalism, which exerted a broadly negative but not homogenizing effect on attitudes toward foreign countries.

This study makes several contributions to the literature as follows. Foremost, it successfully identifies the emergence of exclusionary nationalism and its timing through the analysis of newspaper article data, furthering our understanding of the role of exclusionary nationalism in wars. This new approach to quantitatively capture changes in exclusionary nationalism can be employed in periods and regions where regular opinion surveys are lacking and hence broadens the current empirical basis. Moreover, the rise of exclusionary nationalism in prewar Japan was demonstrated not to uniformly emphasize its superiority over other countries, but rather varying degrees of hostility toward potential adversary and allied nations before conflict. This highlights the potential of disparities in inner- and outer-group perceptions to shape the dynamics of friend–enemy distinctions in times of war.

Detecting early signs of exclusionary nationalism has implications beyond averting the risk of conflicts alone. Exclusionary nationalism exhibits additional linkages to various spheres of society. Notably, it demonstrates a negative correlation with democratic systems [40], indicating its potential to undermine the foundations of inclusive governance. Furthermore, its presence is associated with hindrances to genocide prevention efforts [41], underlining its role in exacerbating ethno-political tensions that can escalate into violent atrocities. Additionally, the adverse effects of exclusionary nationalism extend to resource allocation and public goods provision [42], emphasizing its implications for social cohesion and equitable distribution. Our approach could contribute to refining these causal mechanisms quantitatively based on broader timeframes and geographical regions, which should be examined in the future.

Lastly, we discuss the limitations of our approach and suggest potential avenues for future research. In our method, it is difficult to determine whether exclusionary nationalism was driven by the populace, wealthy elites, or media influencers solely from newspaper articles. This implies a limitation of our approach, which uses newspaper articles during the period leading up to the outbreak of the Pacific War. Conversely, fixed-point public opinion surveys are commonplace today. In addition, in today’s digitally transformed society, the discourse space has shifted online. Thus, future studies should extend the approach employed in our study not only to media coverage but also to fixed-point public opinion surveys and social media platforms. This could contribute to a more comprehensive understanding of current exclusionary nationalism.

This study is also limited by its focus on a single historical case—prewar Japan—and by the observational nature of newspaper data. While the proposed method allows us to detect statistically meaningful shifts in exclusionary nationalist discourse in the Japanese case, it cannot establish whether such shifts generalize across other cases or how they covary with the onset of war in other contexts. Moreover, although the Japanese language allows us to easily distinguish Japanese-origin words from foreign loanwords, this does not hold true for many other languages. However, in the realm of language, etymological dictionaries in many languages record the origin language of each word. Thus, creating such a corpus would be necessary for understanding international relations and tracing linguistic evolution. In fact, recent studies have prompted the active development of multiple etymological databases (see, for example, Refs. [43,44]). Using these databases allows us to distinguish between origin words and foreign loanwords across various languages, enabling us to apply our methodology to other languages in the near future. Thus, future research could extend our approach to multiple countries and conflicts to examine whether similar temporal patterns characterize the relationship between war and exclusionary nationalism more broadly.

## Supporting information

S1 File SI Text. Supplementary information about the dataset and the influence of threshold values of SST on detected change points. (PDF)
