# Supplementary material for: Identifying surge of exclusionary nationalism: A case study of prewar Japan
Source: PLoS One. 2026 Jul 8;21(7):e0349895. doi: 10.1371/journal.pone.0349895 (PMC13345258; doi:10.1371/journal.pone.0349895)
Supplement: S1 File — (PDF) [file pone.0349895.s001.pdf]

Supporting Information

Identifying the Surge of Exclusionary Nationalism: A case study of prewar Japan

Tomoko Matsumoto [1,2], Yutaka Shimada [2,*], Hiroyuki Hirate [3], Tohru Ikeguchi [3]

1 Institute of Arts and Sciences, Tokyo University of Science. 1-3, Kagurazaka, Shinjuku-ku, Tokyo 162-8601, Japan.
2 Graduate School of Science and Engineering, Saitama University. 255 Shimo-okubo, Sakura-ku, Saitama 338-8570, Japan.
3 Department of Information and Computer Technology, Tokyo University of Science. 6-3-1 Niijuku, Katsushika-ku, Tokyo 125-8585, Japan.

* yshimada@mail.saitama-u.ac.jp

S1 The creation of list for *ateji* and *katakana* representations of foreign place names

The following procedures were employed for creating the lists for the *ateji* and *katakana* representations of foreign location names:

- The list of *ateji* representations of a foreign location name was compiled from three sources:
  - *Yonde Tanoshimu Ateji and Nandokugo no Jiten [Dictionary of Ateji and Words Hard to Read]*, edited by Tokyo-Do Publishing Department in 2011 [1].
  - The representations of place names by Fukuzawa Yukichi in *Sekai Kunijin [The World's Nations]*, summarized by the Committee for ateji and loanwords dictionary in 1991, published by Kashiwa Shobo [2].
  - Website "Gaikokumei Gaikoku Chimei Sekai no Toshi no Kanji Hyōki [Kanji Representation of Foreign Names, Foreign Place Names, and World Cities]" from Minna no Chishiki Chotto Benricho (URL: `https://www.benricho.org/kanji/kanji_chimei/` (last visited on April 20th, 2022). According to the website, they created the list using various sources such as the "Gaikoku Chimei Jinmei Shin Jiten [New Dictionary of Foreign Place Names and Personal Names]," published by the Geographic Research Association in 1903.
- The list of *ateji* representations of a place name was created in correspondence with their respective *katakana* representations.
- It is common for multiple *ateji* and *katakana* representations to co-exist for a single place name. For instance, there are three *katakana* representations and seventeen *ateji* representations for Germany (Fig. S1). We recorded all of these.
- For each city name and geographical designation (e.g., the names of lakes, rivers, seas, and mountains), if all representations fall within the territory of a single country, they are organized by country. If the designations span multiple countries, they are recorded as non-country-specific place names.

Katakana representations :

ドイツ　ゼルマニア　ジャーマニー

Ateji representations :

独逸　独乙　独逸蘭土　独国　杜乙蘭土　杜乙　獨逸都　度逸都蘭土　度逸蘭土　日耳曼
入爾馬泥亜　熱爾瑪泥亜　齊尓瑪泥亜　齊尓瑪尼亜　入尓馬泥亜　入耳馬泥亜　入爾耳馬泥亜

Fig S1. Examples of the *katakana* and *ateji* representations for Germany (country name).

S2 The number of monthly newspaper articles in our dataset

To collect newspaper articles from prewar Japan, we contacted Kobe University and utilized the Newspaper Clipping Collections created by their Research Institute for Economics and Business Administration (`http://www.lib.kobe-u.ac.jp/sinbun/e-index.html`). We first selected newspapers with more than 5,000 articles and extracted newspaper articles published in these newspapers, each of which contains 50 or more words. The number of newspaper articles was 269,183, which covers around 90% of the newspaper articles in the newspaper clipping collections provided by Kobe University. Figure S2 illustrates the number of monthly newspaper articles, where the gray solid line shows the original number of monthly newspaper articles and the red solid line shows its moving average over 12 months.

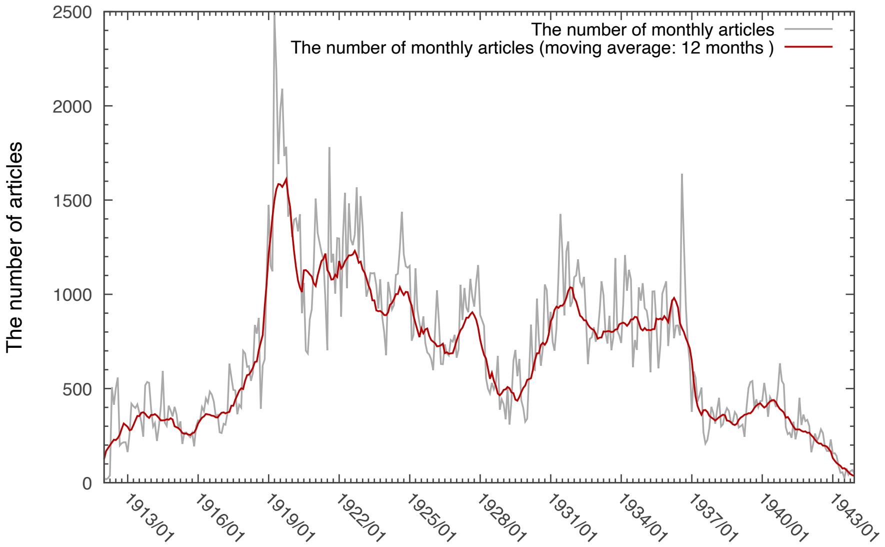

Fig S2. The number of monthly newspaper articles included in the dataset. The gray solid line is the original number of monthly newspaper articles and the red solid line is its moving average over 12 months.

S3 Sensitivity of SST results for the *ateji* ratios to the threshold value

Figure S3(a) illustrates both the *ateji* ratio ($R_{\mathrm{F}}^{\mathrm{A}}(t)$) and the *katakana* ratio ($R_{\mathrm{F}}^{\mathrm{K}}(t)$) in the upper panel, along with the identified change points estimated by the SST in bottom panel. Figure S3(b) and (c) illustrate the enlargements of the bottom panel of Fig. S3(a). In the SST results in Fig. S3, notable peaks are observed in 1924, 1927, 1936, and 1938 when threshold $\theta < 0.4$; the peaks observed in 1924, 1927, and 1936 become smaller with increases in threshold value θ. Because the variation score $\alpha(t)$ is defined as the ratio of the change-point score (CP score) at each time t to the maximum CP score, all values of $\alpha(t)$ for each time point decreases with increase in the threshold value, θ, except for the case where the CP score $c_{M,n}(t)$ is the largest change points in the wide range of parameter values of M and n. This indicates that

the most significant change came in 1938, because the variation score when $t \approx 1938$ is larger than 0.9 for all threshold values. Moreover, the second, third, and fourth changes came in 1927, 1924, and 1936 in the order of the magnitude of $\alpha(t)$. This order holds even when we perform the SST for the *ateji* ratio time series in the time range from June 1912 to June 1938 so that the most significant change observed in 1938 is not included (Fig. S4).

Figures S5(a), S6(a), S7(a), and S8(a) illustrate both the *ateji* ratio ($R_{\mathrm{X}}^{\mathrm{A}}(t)$) and *katakana* ratio ($R_{\mathrm{X}}^{\mathrm{K}}(t)$) for $X \in \{\mathrm{DEU}, \mathrm{GBR}, \mathrm{ITA}, \mathrm{USA}\}$, along with the identified change points estimated by the SST with several threshold values in the bottom panel. From these figures, the detected candidates of pivotal change points are almost the same, even if the threshold values change. Therefore, the discussion and conclusions in the main text apply to these results obtained by various threshold values.

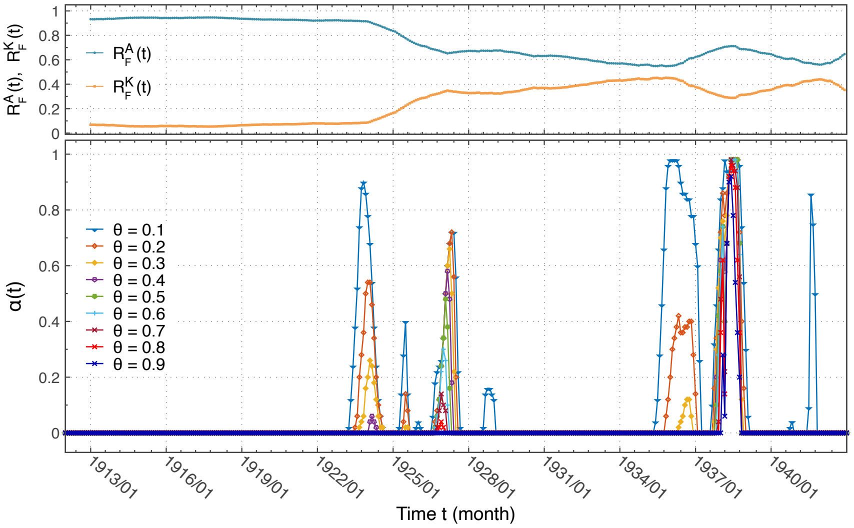

(a)

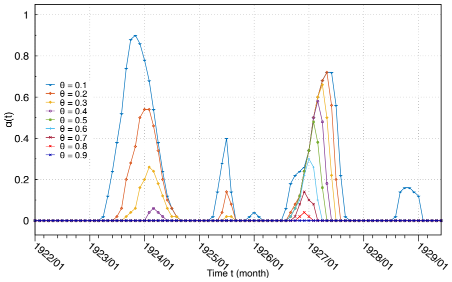

(b)

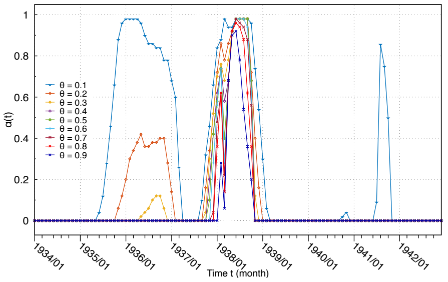

(c)

Fig S3. The *ateji* ratio when the parameter ranges are set to $6 \leq M \leq 12, 6 \leq n \leq 12$, and $M + n \geq 12$. (a) The upper part of the figure shows the ratio of the number of *ateji* words referring to location names in all foreign countries to the number of both *ateji* and *katakana* words referring to them at time t ($R_{\mathrm{F}}^{\mathrm{A}}(t)$). $R_{\mathrm{F}}^{\mathrm{K}}(t)$ ($= 1 - R_{\mathrm{F}}^{\mathrm{A}}(t)$) is also plotted to compare it with the *katakana* ratio. The bottom part of the figure shows the variation score $\alpha(t)$ obtained by the SST. (b) Enlargement of the bottom figure in (a) from January 1922 to February 1929, and (c) enlargement of the bottom figure in (a) from January 1934 to June 1942.

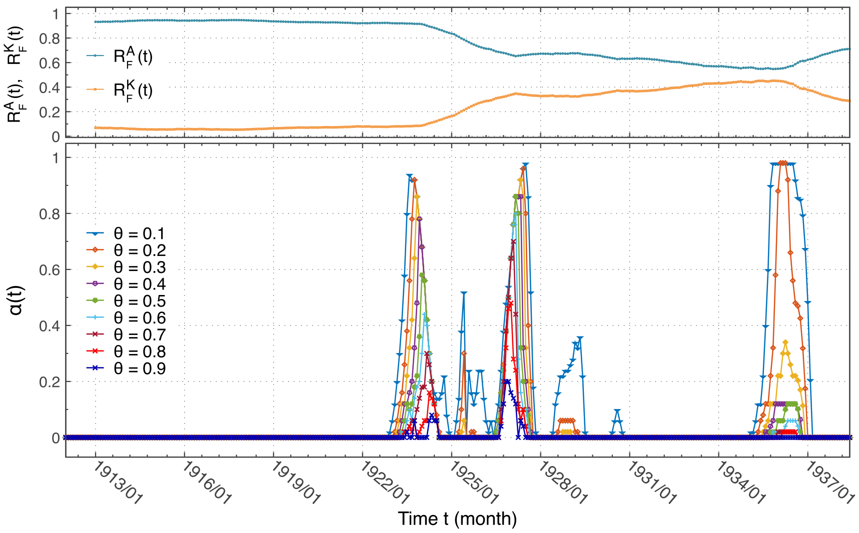

(a)

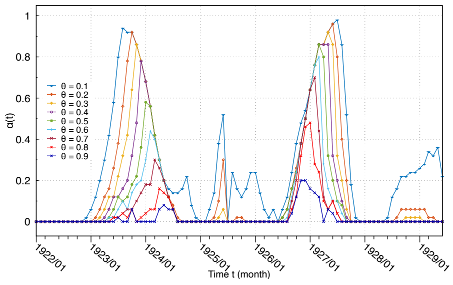

(b)

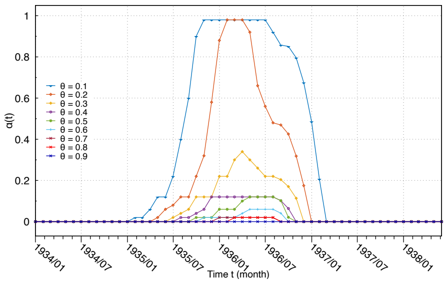

(c)

Fig S4. The *ateji* ratio from June 1912 to June 1938 when the parameter ranges are set to $6 \leq M \leq 12, 6 \leq n \leq 12$, and $M + n \geq 12$. (a) The upper part of the figure shows the ratio of the number of *ateji* words referring to location names in all foreign countries to the number of both *ateji* and *katakana* words referring to them at time t ($R_{\mathrm{F}}^{\mathrm{A}}(t)$). $R_{\mathrm{F}}^{\mathrm{K}}(t)$ $(= 1 - R_{\mathrm{F}}^{\mathrm{A}}(t))$ is also plotted to compare it with the *katakana* ratio. The bottom part of the figure shows the variation score $\alpha(t)$ obtained by the SST. (b) Enlargement of the bottom figure in (a) from January 1922 to February 1929, and (c) enlargement of the bottom figure in (a) from January 1934 to June 1938.

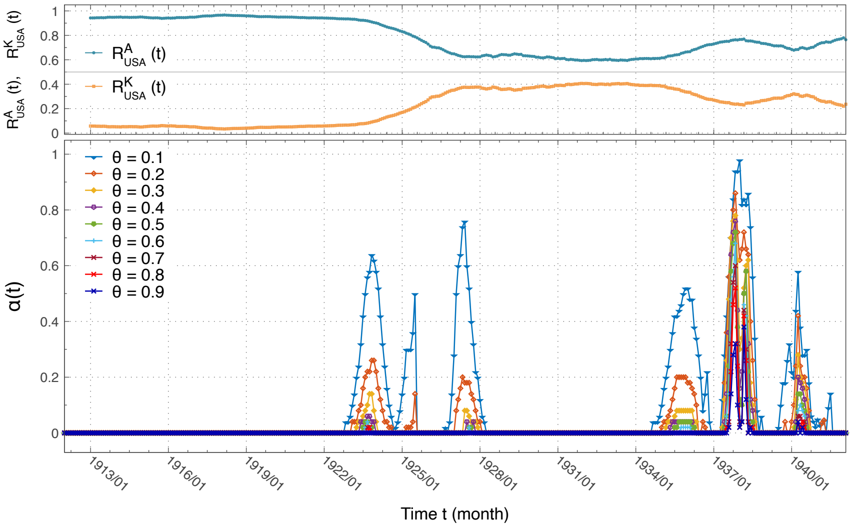

(a)

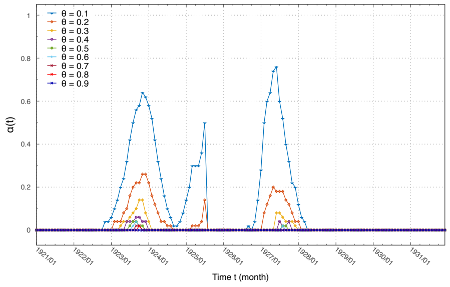

(b)

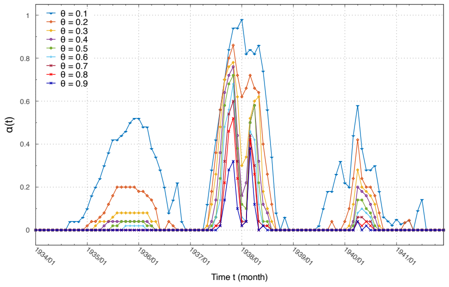

(c)

Fig S5. The *ateji* ratio for USA when the parameter ranges are set to $6 \leq M \leq 12, 6 \leq n \leq 12$, and $M + n \geq 12$. (a) The upper part of the figure shows the ratio of the number of *ateji* words referring to location names in all foreign countries to the number of both *ateji* and *katakana* words referring to them at time t ($R_{\mathrm{F}}^{\mathrm{A}}(t)$). $R_{\mathrm{F}}^{\mathrm{K}}(t)$ ($= 1 - R_{\mathrm{F}}^{\mathrm{A}}(t)$) is also plotted to compare it with the *katakana* ratio. The bottom part of the figure shows the variation score $\alpha(t)$ obtained by the SST. (b) Enlargement of the bottom figure in (a) from January 1922 to February 1929, and (c) enlargement of the bottom figure in (a) from January 1934 to June 1942.

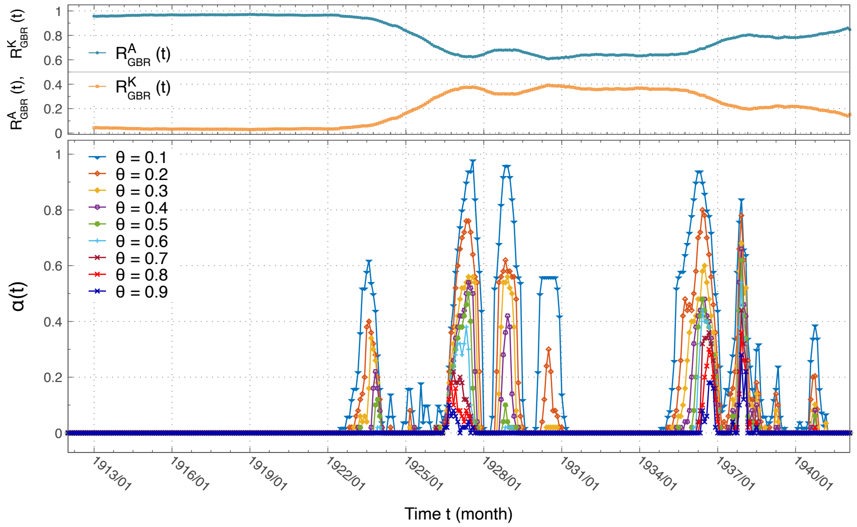

(a)

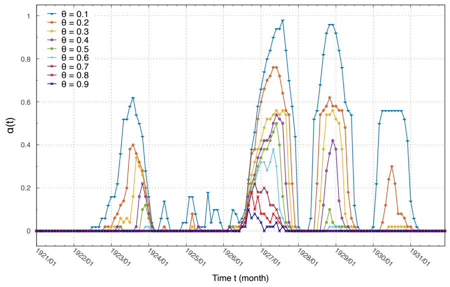

(b)

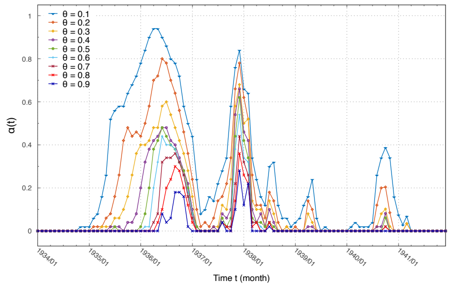

(c)

Fig S6. The *ateji* ratio for GBR when the parameter ranges are set to $6 \leq M \leq 12, 6 \leq n \leq 12$, and $M + n \geq 12$. (a) The upper part of the figure shows the ratio of the number of *ateji* words referring to location names in all foreign countries to the number of both *ateji* and *katakana* words referring to them at time t ($R^{\mathrm{A}}_{\mathrm{F}}(t)$). $R^{\mathrm{K}}_{\mathrm{F}}(t)$ $(= 1 - R^{\mathrm{A}}_{\mathrm{F}}(t))$ is also plotted to compare it with the *katakana* ratio. The bottom part of the figure shows the variation score $\alpha(t)$ obtained by the SST. (b) Enlargement of the bottom figure in (a) from January 1922 to February 1929, and (c) enlargement of the bottom figure in (a) from January 1934 to June 1942.

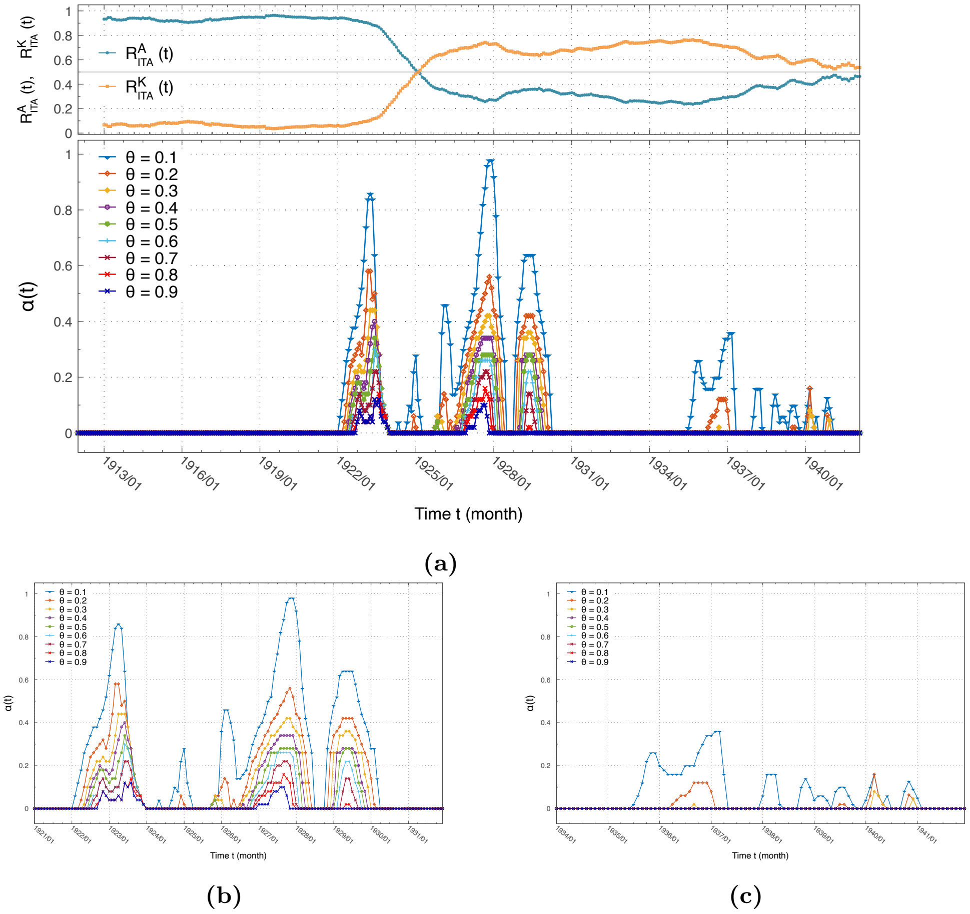

Fig S7. The *ateji* ratio for ITA when the parameter ranges are set to $6 \leq M \leq 12, 6 \leq n \leq 12$, and $M + n \geq 12$. (a) The upper part of the figure shows the ratio of the number of *ateji* words referring to location names in all foreign countries to the number of both *ateji* and *katakana* words referring to them at time t ($R^{\mathrm{A}}_{\mathrm{F}}(t)$). $R^{\mathrm{K}}_{\mathrm{F}}(t)$ ($= 1 - R^{\mathrm{A}}_{\mathrm{F}}(t)$) is also plotted to compare it with the *katakana* ratio. The bottom part of the figure shows the variation score $\alpha(t)$ obtained by the SST. (b) Enlargement of the bottom figure in (a) from January 1922 to February 1929, and (c) enlargement of the bottom figure in (a) from January 1934 to June 1942.

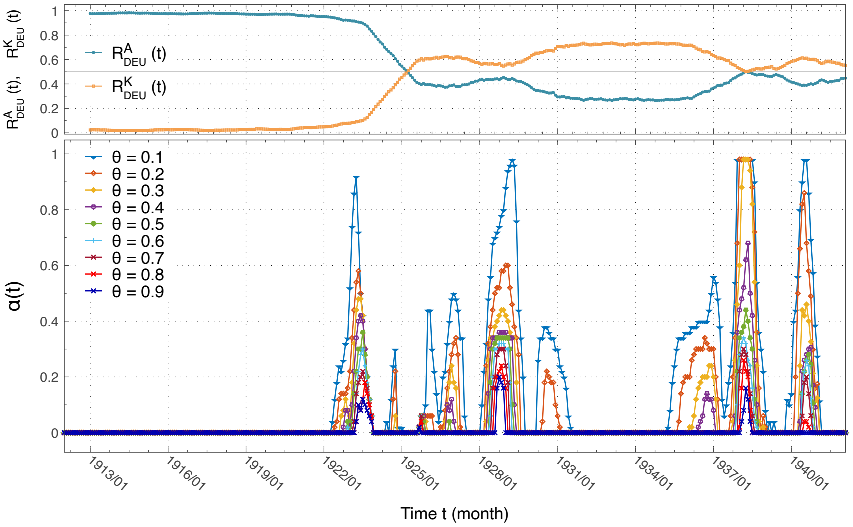

(a)

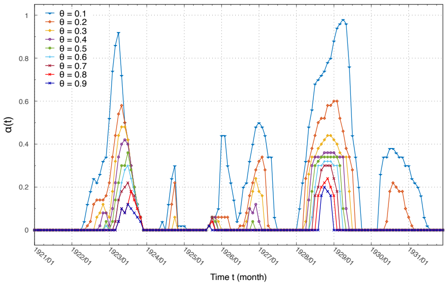

(b)

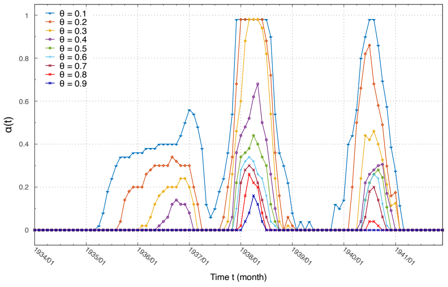

(c)

Fig S8. The *ateji* ratio for DEU when the parameter ranges are set to $6 \leq M \leq 12, 6 \leq n \leq 12$, and $M + n \geq 12$. (a) The upper part of the figure shows the ratio of the number of *ateji* words referring to location names in all foreign countries to the number of both *ateji* and *katakana* words referring to them at time t $(R^{\mathrm{A}}_{\mathrm{F}}(t))$. $R^{\mathrm{K}}_{\mathrm{F}}(t)$ $(= 1 - R^{\mathrm{A}}_{\mathrm{F}}(t))$ is also plotted to compare it with the *katakana* ratio. The bottom part of the figure shows the variation score $\alpha(t)$ obtained by the SST. (b) Enlargement of the bottom figure in (a) from January 1922 to February 1929, and (c) enlargement of the bottom figure in (a) from January 1934 to June 1942.

S4 The *ateji* and *katakana* ratios of country names

We investigated how frequently *ateji* and *katakana* were used to refer to the country names of DEU, GBR, ITA, and USA. Table S1 shows the *ateji* and *katakana* words corresponding to these four countries.

Table S1. The *ateji* and *katakana* words corresponding to names of four countries: DEU, GBR, ITA, and USA, where "DEU," "GBR," "ITA," and "USA" denote Germany, the United Kingdom, Italy, and the United States, respectively.

country	*ateji*	*katakana*
DEU	独逸	ドイツ
GBR	英国	イギリス
ITA	伊太利	イタリア/イタリー
USA	米国	アメリカ

The ratio of the number of *ateji* words referring to a country name, "X", to the number of both *ateji* and *katakana* words referring to it at time t, defined by:

$$\tilde{R}_{\mathrm{X}}^{\mathrm{A}}(t) = \frac{\tilde{P}_{\mathrm{X}}^{\mathrm{A}}(t)}{\tilde{P}_{\mathrm{X}}(t)} \tag{S1}$$

where $\tilde{P}_{\mathrm{X}}^{\mathrm{A}}(t)$ is the number of newspaper articles including *ateji* words referring to country name $\mathrm{X} \in \{\mathrm{DEU}, \mathrm{GBR}, \mathrm{ITA}, \mathrm{USA}\}$ at time t, and $\tilde{P}_{\mathrm{X}}(t)$ is the number of articles including both *ateji* and *katakana* words referring to X at time t. In Eq. (S1), the unit of time is year.

In addition to the *ateji* ratio, we also define the following ratio with respect to *katakana* words:

$$\tilde{R}_{\mathrm{X}}^{\mathrm{K}}(t) = \frac{\tilde{P}_{\mathrm{X}}^{\mathrm{K}}(t)}{\tilde{P}_{\mathrm{X}}(t)} \tag{S2}$$

where $P_{\mathrm{X}}^{\mathrm{K}}(t)$ is the number of articles including *katakana* words referring to country name $\mathrm{X} \in \{\mathrm{F}, \mathrm{DEU}, \mathrm{GBR}, \mathrm{ITA}, \mathrm{USA}\}$ at time t.

We investigated the ratios of country names defined by Eqs. (S1) and (S2) in the three major newspapers with the highest number of articles in the dataset. Figure S9 shows the results for these ratios.

In the Osaka Asahi Shimbun's case (Fig. S9(a)), the *ateji* ratios of enemy countries increase around 1940, while the *katakana* ratios of the ally countries remain higher than the *ateji* ratios. In the cases of the Osaka Mainichi Shimbun and the Chugai Shogyo Simpo (Fig. S9(b) and S9(c)), the *ateji* ratios of the enemy countries are higher than the *katakana* ratios around 1940, while the ratios of the ally countries vary across these two newspapers. In the Osaka Mainichi Shimbun's case (Fig. S9(b)), the *ateji* ratio of Germany remains higher than the *katakana* ratio, and the *ateji* ratio of Italy remains low after around 1925. We would remark that there are two ways to describe "Italy" in *katakana*, which are "イタリー" derived from the English pronunciation of "Italy" and "イタリア" derived from the Italian pronunciation of "Italia."

From Fig. S9(b), around 1940, the *katakana* ratio of "イタリア" increases in the Osaka Mainichi Shimbun's case, and the same applies to the Osaka Asahi Shimbun's case. On the other hand, in the Chugai Shogyo Simpo's case, these *katakana* ratios remain low or decrease around 1940, while the *ateji* ratio increases. Interestingly, the period during which the *ateji* ratios increase or remain higher around 1940 coincides with the eve of the war.

For reference, Table S2 presents examples of articles from the Osaka Asahi Shimbun for the two periods: the 1930s and the period from 1940 onward, where five articles are randomly chosen from each of the periods.

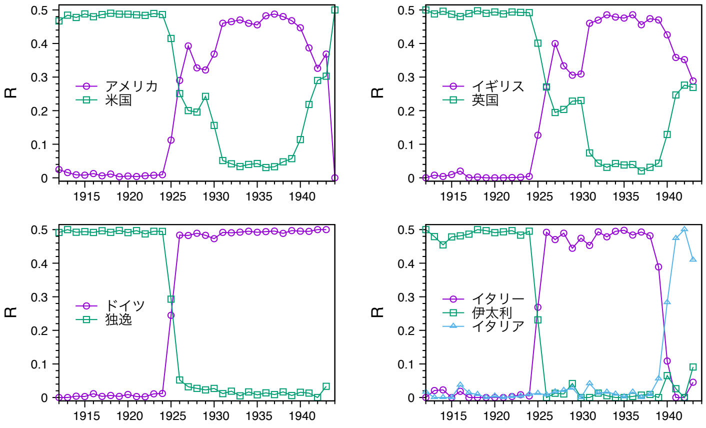

(a) Osaka Asahi Shimbun

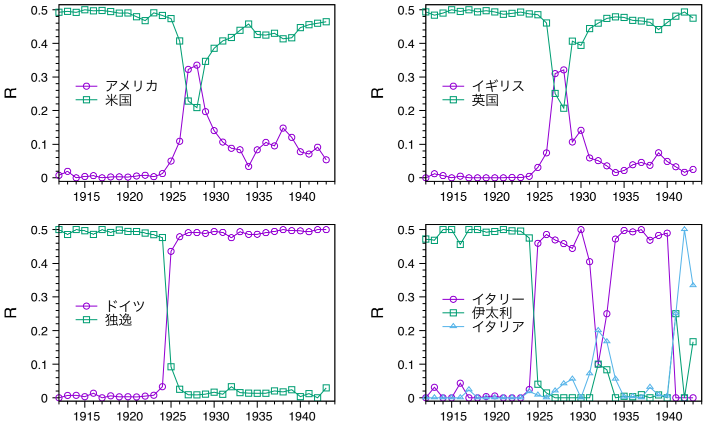

(b) Osaka Mainichi Shimbun

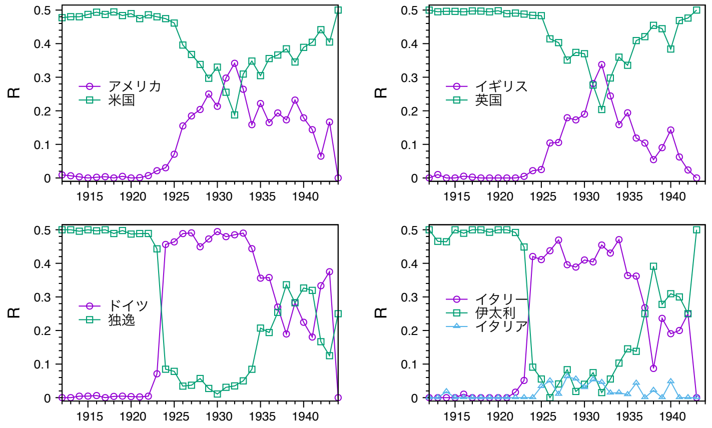

(c) Chugai Shogyo Simpo

Fig S9. Results for the ratios of *ateji* and *katakana* words referring to a country name.

Table S2. Examples of newspaper articles that include the country name of the USA, namely **アメリカ** (highlighted in blue) or **米国** (highlighted in red). In this table, five articles are randomly chosen from the newspaper articles published by the Osaka Asahi Shimbun during two types of periods: the 1930s and after 1940.

Date	Articles (extracted the first approximately 200 characters)
08/14/1931	十四日附夕刊所報の如く米国連邦農事局は棉産地十四州の知事に対し『本年産棉花三分の一 (約五百万俵) を廃棄しては如何、もし各州が同意するならば、政府は現在手持している棉花をもう一ヶ年間売出さぬことにする』旨を提議した　右は米棉大豊作に対するアメリカ政府対策の最初の現われとして注意を喚起し、その実行性如何になお疑問を持ちつつも、市場の人気にはかなり影響が大きかった、何となればこのような政府の提議がなくと . . .
10/29/1932	【ワシントン特電二十七日発】 かつてアメリカ大使として短期間東京に駐在したことのある現国務次官カッスル氏は二十七日オハイオ州クリヴランドにおける或る午餐会の席上一場の演説を試み「アメリカは国際連盟が行動を開始するまでリットン報告に対する論議を差控うべきこと」を再び強調し　左の如く述べた　東洋における問題は他の諸国にとってもアメリカと同様に重大なものであった、そは世界 . . .
01/21/1933	（上）一　日支紛争は国際連盟総会の議に上っているが、これに最も緊切なる利害を持ち、日本が最も重視せねばならぬ国が、却って連盟外のアメリカとロシヤだということは、また何たる奇縁かつ奇観であろう。よって余はここに少しく両国の態度を検して見たい。<br>二　アメリカといえば、明治三十二年秋の支那門戸開放機会均等宣言、三十四年七月支那領土保全通牒、大正十一年二月九国条約の張本人として、支那のために戦うをも辞せ . . .
04/23/1934	【ワシントン二十一日発連合】 アメリカ大統領ルーズヴェルト氏は棉花生産制限と超過棉花に対する課税を規定したバンクヘッド法案に署名これを裁可した、同案の主要点は左の通り、一、新棉の生産高を一千万俵に限定す　一、右割当以上の生産棉に対しては中央市場の市価平均の五割に相当する課税を行い棉花販売の際にこれを課す　一、本法施行期間は一ケ年とし農家三分二がその延長を要望する場合はさらに一ケ年延長することを得 . . .
08/18/1935	太平洋が漸く多事となるに従いパナマ運河の軍事的価値はますます重大となって来た、それがためアメリカ合衆国とパナマ共和国との関係も面倒になって来た結果、両国の間に新条約締結の交渉が開始されていた、しかして今回いよいよ基本条約締結に関し意見一致したという、これは両国の関係を改善する上に大いに貢献するところがあろう、現在の条約は一九〇三年のパナマ革命後新設のパナマ共和国とアメリカ合衆国との間に締結され . . .
11/16/1940	英国政府は今回突如として英国の極東軍を拡大統合し、その新司令官に陸軍の大物サー・ロバート・ブルック＝ポッパム空軍大将を任命し、その司令本部をシンガポールにおくことに決定した、英本国はいまや連日にわたるドイツ空軍の猛爆に対し必死の防衛戦を続けているが、持久戦継続の頼みの綱は米国の援助と自治領および植民地の協力一致である、わけてもインド、ビルマ、濠洲、マレーなど極東における宝庫の維持は刻下の急務で . . .
11/18/1940	【ニューヨーク特電十七日発】 米下院の反米運動調査委員会ダイズ委員長は十六日日独伊およびソ連の四ヶ国が米国の軍拡とその対英援助を妨害しつつあり、近くその証拠を纏めた報告書を発表する旨を言明した、ダイズ委員会ははじめこの報告書を発表でなく独伊両国領事館の米国における活動につき公聴会を開催する予定であったが、その席上におけるダイズ氏の不用意の言動を口実にドイツが報復手 . . .
08/05/1941	【マニラ特電四日発】 輸出許可制の実施による対日貿易の激減とアメリカ船舶のフィリッピン航路引揚げがまき起した船腹不足のため未曾有の経済難局に立った比島政府では来る二十三日のクリッパー機でロハス蔵相をワシントンに特派し対アメリカ輸出税の一時停止、対アメリカ輸出数量制限の緩和、アメリカ船舶の回航および金融援助の交渉を行わせることになった模様である、フィリッピン . . .
01/01/1942	眼下に見る修羅場!紀元二千六百一年十二月八日わが海軍航空部隊をはじめとする奇襲部隊は朝霧の真珠湾に突撃を敢行、米国太平洋艦隊は忽ちにして全滅した、元旦の紙面を飾るこのハワイ海戦の写真はいずれも空襲に参加した海鷲の手によって撮影されたもの、われわれもまた荒鷲とともにこの大空襲戦を眼 . . .
10/06/1942	【イスタンブール特電五日発】従来英国が独占していたイラク、イランの石油会社は最近米資本の侵入著しくイラン石油会社の英持株の九割は米資本に吸収され、また目下テヘランにおいて英イラン石油会社の株式譲渡の交渉が米資本家代表と会社側代表との間に行われつつあり、西亜における米国の軍事的進出は宣伝ほど目ざましくはないが、政治的もしくは経済的進出は最近とみにに拡大され、同方面における英国の地位は第二位的なもの . . .

References

1. Tokyo do publishing department, editor. *Yonde Tanoshimu Ateji and Nandokugo no Jiten [Dictionary of Ateji and Words Hard to Read]*. Tokyo: Tokyo-do; 2011.

2. Committee for ateji and loanwords dictionary, editors. *Ateji Gairaigo Jiten[Dictionary of ateji and lanwords]*. Tokyo: Kashiwa shobo; 1991.
